# ORIGINS: Nutritional Profile of Children Aged One Year in a Longitudinal Birth Cohort

**DOI:** 10.3390/nu17091566

**Published:** 2025-05-01

**Authors:** Sarah Whalan, Poonam K. Pannu, Rachelle A. Pretorius, Alexander J. J. Scherini, Sonia Gregory, Susan L. Prescott, Desiree Silva

**Affiliations:** 1 The Kids Research Institute Australia, Perth Children’s Hospital, Nedlands 6009, Australia; poonam.pannu@thekids.org.au (P.K.P.); rachelle.pretorius@thekids.org.au (R.A.P.); alex.scherini@thekids.org.au (A.J.J.S.); sonia.gregory@thekids.org.au (S.G.); susan.prescott@thekids.org.au (S.L.P.); desiree.silva@thekids.org.au (D.S.); 2 School of Medical, Molecular and Forensic Sciences, College of Environmental and Life Sciences, Murdoch University, Murdoch 6150, Australia; 3 School of Medical and Health Sciences, Edith Cowan University, Joondalup 6027, Australia; 4 Faculty of Science, Medical School, University of Western Australia, Crawley 6009, Australia; 5 Department of Immunology, Perth Children’s Hospital, Nedlands 6009, Australia; 6 Department of Paediatrics and Neonatology, Joondalup Health Campus, Joondalup 6027, Australia

**Keywords:** ORIGINS, nutrition, cohort, early childhood, DOHaD

## Abstract

Background: Dietary intake during the first year of life is a key determinant of a child’s growth and development. ORIGINS is a longitudinal birth cohort study investigating factors that contribute to a ‘healthy start to life’ and the prevention of non-communicable diseases. Methods: This descriptive cross-sectional study aims to describe the dietary intakes of one-year-old children participating in ORIGINS and compare these to the Australian Dietary Guidelines and Nutrient Reference Values (NRVs). Between 2020 and 2023, dietary intake data were collected on 779 one-year-old children using a Food Frequency Questionnaire (FFQ). The analysis explored milk intake (breastmilk, infant formula, and cow’s milk), the introduction to solids, macronutrient, micronutrient, and food group intakes. Results: The results indicated that 41.5% were still being breastfed at one year of age, while 58.0% continued to receive formula milk. While the cohort met NRV cut-offs for most micronutrients, iodine intake fell below requirements, and sodium intake exceeded recommendations. Diet quality, based on the food group intake, did not meet recommendations, with children over-consuming fruit and discretionary foods, while under-consuming vegetables and cereals and grains foods. Conclusions: These findings highlight areas for improvement in the dietary intake of one-year-old children.

## 1. Introduction

The first year of life is a time of extensive growth and development, making it essential to meet infants’ nutrient demand [1,2,3]. For the first four to six months, breastmilk or infant formula milk adequately meets nutritional requirements; however, at around six months, complementary (solid) foods become necessary to meet the increased energy and nutrient needs for growth and development [1,2,3]. This transition from milk-based to solid foods is crucial for ensuring that young children receive adequate macronutrients and micronutrients to meet their growing nutritional requirements [3,4]. 

Poor dietary intake may lead to nutrient deficiencies, affecting various growth and developmental milestones during early childhood [5]. Insufficient macronutrient intake can negatively affect key neurodevelopmental processes and has been associated with lower IQ scores and increased behavioural dysregulation [5]. Micronutrient intake is also critical for cognitive, motor, and socioemotional development [5,6,7,8]. Deficiencies in iron, iodine, zinc, and vitamin A have been linked with impaired developmental outcomes in young children [6]. Furthermore, failure to meet energy and nutrient requirements may lead to growth faltering, cognitive impairment, and increased risk of infection [9,10,11]. 

Assessing early dietary behaviours in young children presents significant challenges [8]. Among children aged one to three years, diets are highly variable, with rapidly changing food habits and transitions in dietary patterns. Additionally, accurately reporting how much food is offered versus what is consumed is difficult to capture [8]. In Australia, there is little known about the dietary intake of children under the age of two, as this age group has been excluded from national nutrition surveys [12,13,14]. There have only been a small number of studies within Australia that have reported on dietary intake in this age group, most of which are over 10 years old [15,16,17,18]. Most recently, Moumin et al. [9] assessed the energy and nutrient intake in a national sample of 1140 Australian children aged 0–24 months using a one-day food record. This study, along with a select few studies that have examined energy and nutrient intake in children less than two years of age, consistently identify certain micronutrient deficiencies, particularly in iron and zinc, and excessive intake of sodium [9,15,16,17,18].

Understanding the dietary intake of young children requires tailored assessment tools that can be administered at the population level, such as Food Frequency Questionnaires (FFQs). A recent Australian study, Infant Feeding Activity and Nutrition Trial (InFANT), demonstrated good to acceptable validity of their short food frequency questionnaire compared to three non-consecutive 24-h dietary recalls for assessing certain nutrients (e.g., protein, fat, fibre, iron, vitamin C, folate) and food items (e.g., water, eggs, potatoes, hot chips, yoghurt) [19]. The FFQ used in this study has been specifically designed and validated [19] to assess dietary intake in young children.

Contemporary data on population intakes are required in order to address the gaps in dietary data for young children and to determine whether the dietary patterns meet national dietary guidelines. Therefore, this descriptive cross-sectional study aimed to describe the dietary intake of one-year-old children in Western Australia participating in the ORIGINS longitudinal birth cohort study [20]. The validated short FFQ developed as part of InFANT [19] was used to assess dietary intake in the ORIGINS cohort, which was then compared to the Nutrient Reference Values for Australia [21]. Additionally, this study reports on breastfeeding, formula feeding, cow’s milk intake, and introduction to solids in this cohort. This research contributes valuable insights into early childhood nutrition and highlights areas for potential dietary improvements. 

## 2. Materials and Methods

### 2.1. Study Design and Participants 

This descriptive cross-sectional study is nested within ORIGINS [20], which is a unique longitudinal study conducted in collaboration with The Kids Research Institute Australia and Joondalup Health Campus (JHC). ORIGINS is one of the most comprehensive studies of pregnant women and their families in Australia to date, recruiting 10,000 families over a decade from the Joondalup and Wanneroo communities in Perth, Western Australia (WA). It is a prospective birth cohort study that includes both observational and interventional components. Further details on the ORIGINS study protocol have been previously published [20]. 

Participant recruitment for ORIGINS took place at JHC, Perth, WA. All pregnant women planning to deliver their baby at JHC, from both the public and private hospitals, were eligible to enrol in the study at one of three timepoints during their pregnancy: 20 weeks, 28 weeks, and 36 weeks’ gestation. Participants could enrol as an ‘active participant’ which includes in-depth self-reported surveys, clinic visits, sample collections, and routine hospital and linked data, or as a ‘non-active participant’, which includes routine hospital and linked data only. Written informed consent was obtained from all study participants, with a member of the ORIGINS research team ensuring they had a thorough understanding of the study. The mother then provided consent on behalf of her infant(s) at birth.

Participants are followed up at multiple timepoints, including: 36 weeks’ gestation (if enrolled at 20 or 28 weeks’ gestation), birth, 2 months, 6 months, 9 months, 1 year, 2 years, 3 years, 4 years, and 5 years. This descriptive cross-sectional study focuses on the children at the one-year follow-up. 

### 2.2. Ethics

Ethics approval was granted by the Human Research Ethics Committee of JHC, Ref. 1440 on 16 September 2016. The research related to human use complied with all the relevant national regulations and institutional policies and was performed in accordance with the tenets of the Helsinki Declaration and has been approved by the authors’ institutional review board or equivalent committee. Informed consent was obtained from the birthing mother antenatally, and she then provided consent on behalf of the child once they were born. The research conducted is not related to animal use.

### 2.3. Demographic, Breastfeeding, Formula Feeding and Introduction to Solids Data 

An online survey, the ORIGINS Core Questionnaire, was completed by the ORIGINS birth mother to obtain demographic data, including education level, ethnicity, employment history, marital status, and household income, along with information related to medical history and lifestyle factors following the participant’s enrolment into ORIGINS. Maternal education was categorized as ‘high school or lower’, ‘trade/ apprenticeship/ diploma’, or ‘University degree’. Maternal employment status was categorized as ‘parent/ home duties/ unemployed’, ‘employed full-time (paid or unpaid)’, ‘employed part-time (paid or unpaid)’, ‘student’, or ‘other’. Household composition was separated into the number of adults and the number of children in the household.

The ORIGINS child(ren), along with a caregiver (i.e., birth mother, birth father, grandparent, etc.), attended a paediatric health check between one and one and a half years of age. As part of this health check, the child’s anthropometrics were measured in light clothing by trained staff. Weight was measured to 0.1 g using calibrated infant digital scales. Height was measured to 0.1 cm using a fixed and calibrated stadiometer twice, and if the two measures differed by more than 1.0 cm, a third measurement was taken. 

During this health check, data were collected by the paediatrician on breastfeeding and/or formula feeding, including duration or continuation. Participants were also asked to indicate what age the child was introduced to solids and what specific foods the child had been exposed to.

### 2.4. Food Frequency Questionnaire 

A short, 68-item Food Frequency Questionnaire (FFQ) [19], which collects the child’s usual dietary intake, was completed by the primary caregiver via an online survey when their child reached one year of age. The FFQ used as part of this study has been developed and validated against three non-consecutive 24-h dietary recalls by Zheng et al. [19] and uses a food list informed by the 2007 National Children’s Nutrition and Physical Activity Survey (NCNPAS). The FFQ is comprised of two sections: The first section contains questions relating to general eating habits, including supplement use, while the second section assesses the frequency of consumption of 68 food items over the previous month with nine response options (e.g., ‘never or less than once a month’ through to ‘6 or more times a day’) [19].

The food and nutrient intakes from the FFQ have been calculated using a database incorporating the AUSNUT2007 food composition database. This database was developed by matching each of the 68 food items to one or more foods from AUSNUT2007 [19]. The database then converts the frequency of consumption data into the amount of each food item consumed in grams and then calculates the nutrient intake for each participant.

### 2.5. Nutrient Reference Values (NRVs) 

Nutrient Reference Values (NRVs) have been developed by the National Health and Medical Research Council of Australia as a set of daily nutrient intake recommendations based on the current scientific knowledge [21]. The estimated nutrient intakes for each participant were compared to these NRVs for the life stage “children aged 1–3 years” to determine whether the children in ORIGINS were meeting or not meeting the recommendations at the age of one year. To estimate the prevalence of inadequate and excessive nutrient intakes, the estimated energy requirements (EER), estimated average requirement (EAR), adequate intake (AI), and tolerable upper limits (UL) described in the NRVs for Australia and New Zealand [21,22] were used. 

### 2.6. Statistical Analysis

Descriptive statistical analyses were undertaken using Stata (16.1, StataCorp LLC, College Station, TX, USA). Categorical data are presented using frequencies and proportions. Continuous data are described using means and standard deviations, or medians and interquartile ranges when data are skewed.

Each of the food items collected by the FFQ was categorized into the Australian Guide to Healthy Eating five food groups (fruits, vegetables, cereals and grains, dairy, and meats and alternatives) or identified as a discretionary food [22]. Discretionary foods are higher in fat, added sugars, and salt, and are not recommended for young children [22].

Food group totals were calculated and examined to identify implausible values for this age group. A participant was excluded from the analysis for the following: (1) the fruit food group if the reported daily consumption of fruits was greater than 1000 g; (2) the vegetable food group if vegetable intake was more than 400 g per day; (3) the cereals and grains food group if the reported daily intake was greater than 600 g; (4) the dairy food group if the reported daily intake was more than 1200 g; (5) the meats and alternatives food group if consumption of these foods was greater than 500 g per day; and (6) the discretionary food group if the daily intake was more than 200 g.

The daily number of servings of each of the five food groups was calculated for each child using the amount of food consumed and the standard serving sizes found in the Australian Dietary Guidelines [22]. For rice, pasta, and noodles, the serving size in these guidelines is given as a range of 75–120 g, and the midpoint of this (97.5 g) was used in the calculations. Since there is no allowance for discretionary foods in the recommended dietary patterns for children younger than two years, it is unclear if the serving size for discretionary foods (600 kJ) applies to this group [22]. For this reason, we reported a smaller serving size of 418 kJ/serving, consistent with toddler snacks [1].

Under- or over-reporting of energy intake was determined using the ratio of reported energy intake (EI) and the 12-month-old gender-specific EER [21]. Daily energy intake was considered plausible if the ratio of EI:EER was between 0.54 and 1.46 [9,18,23]. Participants with energy intakes outside of this range were excluded from the macronutrient and micronutrient analyses, as were those with implausible values for one or more of the food group totals.

Macronutrient and micronutrient intakes were compared to the NRVs, including the EER for children aged 12 months, EAR for children aged 1–3 years, AI for infants aged 7–12 months (fat and carbohydrate total) or 1–3 years (fibre, sodium, and potassium), and the UL for children aged 1–3 years [21,22]. The proportions of the sample with inadequate and excessive intakes were calculated using the EAR and UL for 1–3-year-olds. For those nutrients that lack an EAR, the proportion of participants meeting the AI is reported [21,22]. The AI for sodium is assumed to be 400 mg in determining the compliance proportions. The UL listed for folate is for intake from fortified foods and supplements such as folic acid, and the UL listed for magnesium refers to supplements.

The FFQ did not collect any data on breastfeeding; therefore, the analysis does not take into consideration any nutritional contribution from breastmilk. To account for the relatively high breastfeeding rate in this cohort at one year of age, the daily macronutrient, micronutrient, and food group intakes are stratified according to breastfeeding status at the time of the FFQ.

## 3. Results

### 3.1. Participant Characteristics

Between October 2020 and March 2023, FFQ data were collected from 779 children from 774 pregnancies (755 mothers). A total of 42 participants were excluded from at least one food group analysis and were also excluded from the macronutrient and micronutrient analyses. Additionally, 199 participants were excluded from the macronutrient and micronutrient analyses due to implausible energy intakes. As a result, 538 children were included in the macronutrient and micronutrient analyses (Supplementary Figure S1).

The demographic and anthropometric characteristics of the mother–child dyads are presented in Table 1. The age of children at the one-year time point ranged from 10.9 months to 16.4 months, with a mean age of 12.3 months. A total of 51.4% of the children were male, and 34.0% were attending childcare. The mean birth weight was 3433 g, the mean birth length was 50.6 cm, and children’s current mean weight and length were 10.0 kg and 75.0 cm, respectively. 

At the time of enrolment in ORIGINS, the mothers of these children were a mean age of 32.1 years. The majority (83.9%) were of Caucasian ethnicity, 56.8% were born in Australia, and 58.1% held a university degree. The median pre-pregnancy body mass index (BMI) of these women was 24.6 kg/m^2^. When their child was one-year-old, 40.1% were employed on a part-time basis. Most households consisted of two adults (87.7%) and one child (58.8%).

### 3.2. Breastfeeding, Infant Formula Feeding, and Cow’s Milk Intake

The breastfeeding, infant formula feeding, and cow’s milk intake of the ORIGINS children at one year of age are presented in Table 2. While 93.8% of children had been breastfed, only 21.8% had been exclusively breastfed for the first six months. At one year of age, 41.5% were still being breastfed. In comparison, 79.3% of children had received infant formula milk, with 58.0% of children still consuming formula milk at one year of age. Additionally, 43.4% of participants had consumed cow’s milk, separately from cow’s milk formula, by their first birthday. Of those children who had consumed cow’s milk, on average, they were having one feed (serve) per day.

### 3.3. Introduction to Solid Foods 

A summary of the ORIGINS children’s introduction to solid foods is presented in Table 3. Nearly all (99.7%) children were introduced to solids by one year of age, with the majority starting at or after six months (89.2%). At one year of age, 89.7% of children were eating all the food that the caregiver was consuming, and 11.2% were reported as a “fussy eater” by their caregiver. Most children (greater than 90%) had been exposed to high-allergen foods, including eggs, cow’s milk, wheat, fish, and peanuts by one year; however, only 51.6% had been exposed to cashew nuts. An allergy to one or more foods was reported in 8.6% of children. 

### 3.4. Macronutrient Intake

The mean daily energy and macronutrient intake for one-year-old children, stratified by breastfeeding status at the time of the FFQ, is presented in Table 4. Children who were non-breastfed met the EER for both males and females (mean of 3655 kJ/day and 3553 kJ/day, respectively). However, children who were breastfed did not meet the EER for either males or females (3457 kJ/day and 3122 kJ/day, respectively). The mean daily intake for protein exceeded the EAR of 12 g/day by threefold in both the breastfed and non-breastfed groups. The mean daily intake for total fat fell below the AI of 30 g in both the breastfed and non-breastfed groups; however, over a quarter of the children (25.5% breastfed and 43.8% non-breastfed) consumed more than the AI for fat per day. Similarly, the mean daily intake for fibre fell below the AI of 14 g in both the breastfed and non-breastfed groups; however, many of the children (38.5% breastfed and 24.4% non-breastfed) consumed more than the AI for fibre per day. The mean daily intake for total carbohydrate was greater than the AI of 95 g in both the breastfed and non-breastfed groups, with at least half (52.7% breastfed and 65.2% non-breastfed) consuming more than the AI for carbohydrate per day. 

### 3.5. Micronutrient Intake

The median daily micronutrient intake for one-year-old children, stratified by breastfeeding status at the time of the FFQ, is presented in Table 5. The breastfed children, on average, were more likely to consume less than the EAR for several of the micronutrients, including folate (20.1% of children), calcium (42.3%), iron (17.6%), and iodine (75.3%). The median daily intake for iodine fell within the EAR of 65 mg for the non-breastfed group; however, 48.8% of these participants consumed less than the EAR for iodine per day. The median daily intake for sodium exceeded the AI of 200–400 mg for both the breastfed and non-breastfed children. 

### 3.6. Food Group Intake

The median daily intake and servings of each of the five main food groups and discretionary foods for one-year-old children, stratified by breastfeeding status at the time of the FFQ, are presented in Table 6. The only food group that was different between the breastfed and non-breastfed children was dairy foods; those who were currently breastfed consumed only 125 g per day (0.9 serves/day), compared to 431 g per day (1.9 serves/day) consumed by the non-breastfed group. Median daily servings for fruit were exceeded in both the breastfed and non-breastfed groups, while dairy foods were exceeded in only the non-breastfed group. Lean meat and poultry recommendations were being met, while vegetables and cereals and grains foods were below the recommendations for both groups. Children in both the breastfed and non-breastfed groups were reported to be consuming discretionary foods, which exceeds the recommendation of no servings per day for this age group. 

## 4. Discussion

This study is one of the few in Australia to examine the dietary intake of children as young as one year of age and compare it to the Australian Dietary Guidelines and NRVs [9,24]. The cohort met NRV cut-offs for most micronutrients, except for iodine in the breastfed group (below the EAR) and sodium in both the breastfed and non-breastfed groups (exceeding the AI). Children did not meet the dietary guidelines for food groups, over-consuming fruit and discretionary foods, while under-consuming vegetables and cereals and grains foods.

Of the studies that explore the dietary intake of young children, only two used the FFQ to collect dietary intake from the children. One such study, conducted by Marriot et al., [25] in the UK, reported a mean energy intake of 4422 kJ/day in a cohort of one-year-old children. However, this study included breastfed participants without stratifying the results due to the small number of breastfed participants [25]. The study also reported a higher mean intake for total carbohydrates (135.0 g), total fat (42.4 g), retinol (569 µg), and calcium (877 mg) and a lower median intake of vitamin C (79.6 mg) and folate (143 µg) in their cohort compared to the nutrient intake in the ORIGINS children. Another study conducted by de Souza et al. [26] in Canada reported a mean intake of 1991 kcal/day (8330 kJ/day) in a cohort of one-year-old children who were non-breastfed, which is much higher than the mean energy intake in our cohort, which was between 3122 kJ/day (female, breastfed children) and 3655 kJ/day (male, non-breastfed children). The study undertaken by de Souza et al., [26] also reported higher nutrient intake values compared to the values reported in our cohort, including mean total fat (77 g), mean total carbohydrates (259.5 g), median folate (279 µg), median retinol equivalents (741 µg), median calcium (1488 mg), median sodium (1568 mg), and median potassium (2825 mg). This large difference may be attributed to the fact that participant data in ORIGINS were excluded from analysis if their nutrient intake values were considered implausible for a one-year-old (i.e., an EI:EER ratio that fell outside a range of 0.54–1.46).

Exclusive breastfeeding provides all the essential nutrients and antibodies required for optimal infant growth and development, particularly in the first six months of life [27]. In our study, 21.8% of infants were exclusively breastfed until six months of age. This rate is lower than the 37.5% of infants exclusively breastfed at six months, as reported in the National Health Survey 2022 [28]. A potential explanation for the difference in rates could be that, in high socio-economic societies, mothers may return to work earlier, which may influence breastfeeding rates. The mothers in the ORIGINS cohort were of high SES, with over 50% having returned to work on a part-time or full-time basis by one-year post-birth. At one year of age, 41.5% of mothers in our study were still breastfeeding their children, a figure similar to the National Health Survey 2022, where 43.0% of children received breastmilk at one year of age [28]. 

Due to the high breastfeeding rate in this cohort at one year of age, the daily macronutrient, micronutrient, and food group intakes were stratified by breastfeeding status at the time of the FFQ. Overall, the children who were breastfed had lower intakes of most nutrients, including lower energy intake for both males and females, and calcium, iodine, and iron intakes compared to the non-breastfed group. This was to be expected, as the FFQ analysis did not capture breastmilk intake as a source of nutrient intake [27]. In addition, the breastfed group consumed fewer serves per day of dairy foods (125 g/day) compared to the non-breastfed group (431 g/day). This difference may be due to breastfed children receiving adequate nutrition from breastmilk, leading to lower consumption of additional dairy products in the FFQ. For the same reason, breastfed children had lower reported calcium intake. It can be assumed that this group would meet recommendations for the dairy food group if breastmilk were included as a source.

Non-breastfed children consumed above the recommended requirements for energy intake (males 3655 kJ/day vs. EER 3500 kJ/day; and females 3553 kJ/day vs. EER 3200 kJ/day), protein (41.7 g vs. EAR 12 g), and carbohydrate (103.9 g vs. AI 95 g). However, their fat (28.7 g vs. AI 30 g) and fibre intake (11.3 g vs. AI 14 g) were below the recommended requirements. The lower fibre intake could be attributed to the reduced intake of cereals and grains and vegetable intake seen in this cohort, which is similar to other studies investigating dietary patterns in young children [9,15]. Fat levels were close to meeting the AI, which is also similar to other studies [9,15].

Protein intake was more than threefold above the EAR in both the breastfed and non-breastfed groups. Interestingly, lean meat and poultry intake met the recommendations in this cohort, which contrasts with findings from another study in Australia exploring dietary intake of toddlers [1]. Protein and iron intake generally fell within the recommendations in our cohort, which is not consistent with other studies [9,15,16,17,18]. This discrepancy may be due to an overestimation of lean meat and poultry consumption by the caregiver, which subsequently increases the estimation for other nutrients, such as protein and iron. In addition, the FFQ itself did not ask the caregiver to report portion size; rather, the nutrient intake was calculated using median portion size for the food item based on the age of the child. This is likely to have overestimated the intakes in general, a limitation reported by Zheng et al. [19] as part of their validation study using this FFQ. Furthermore, consuming solid foods at or after six months (89.2%) and cow’s milk introduction (43.4%), separate from cow’s milk infant formula by one year of age, may overestimate macronutrient intakes.

Much like other studies exploring the dietary intake of young children in Australia, the children in this cohort exceeded the recommendations for fruit and discretionary foods, while not meeting recommendations for vegetables and cereals and grains foods [9,15]. This dietary pattern places the children at greater risk of micronutrient deficiency, which in turn can negatively affect key neurodevelopmental processes [5]. The provision of foods at the recommended amounts from the five food groups ensures children receive enough nutrients essential for health, growth, and development [21].

In Australia, the commercial toddler food market, particularly products marketed as healthy snacks, may contribute to the overconsumption of discretionary foods in young children’s diets [9,29]. These types of snacks are particularly high in fat and salt and, as such, should be consumed in only small amounts [21]. Discretionary choices are named as such, as they are not considered an essential part of the dietary pattern [21]. Sodium intake was excessive in this cohort, similar to other studies examining dietary intake in children under two years of age [9,15,16,17,18]. There is a potential for this early exposure to increase their preference for salty foods later in life [30]. In later life, a high salt intake is a risk factor for hypertension and cardiovascular disease [21].

### Strengths and Limitations

Only one other study has examined the dietary intake of young children in Western Australia. The ORIGINS study offers a valuable snapshot of the dietary intake of this population group, along with the dietary habits of their mothers across multiple timepoints. The longitudinal design of ORIGINS enables researchers to examine dietary intakes across timepoints to identify trends over time. In the future, a longitudinal comparison of the results at one year with those at three years and five years of age will help identify potential shifts in the dietary intake of young children. Additionally, the cohort study design facilitates the collection of extensive demographic data, which can support comparisons with other studies. Our study is one of few to stratify the results by breastfeeding status.

When completed correctly, a validated FFQ provides an assessment of the habitual dietary intake of an individual. FFQs are commonly used in population-based studies due to cost efficiency and ease of administration. However, self-reported dietary measures are subject to measurement error [31], meaning the estimates may not reflect actual intake. In our study, incorrect completion of the short FFQ, including extreme values and inaccuracies in reporting, such as under- and over-reporting of dietary intakes, resulted in the exclusion of approximately 200 participants. The accuracy of dietary assessment in young children depends on the caregiver’s ability to reliably report intake [8]. This includes potential underreporting of food wastage, and the quantity of meals consumed [24]. Since the FFQ does not capture portion sizes, foods consumed in small quantities may be overestimated, while those consumed in larger amounts may be underestimated. Caregivers may also over-report intake to portray their child as eating well, especially in an age marked by fussy eating and neophobia [24].

To our knowledge, there are no published studies that have evaluated the sensitivity and specificity of this short FFQ in identifying children with inadequate or excessive intakes. There is a need for future studies to assess these metrics in the context of nutrient adequacy in children. A new tool that may be able to more accurately collect dietary intake data is an image-based dietary assessment tool. This involves food images being captured by a handheld device or camera and could improve the accuracy of traditional dietary intake methods [32].

In addition, we found that the FFQ does not capture the composition or nutrient content of breastmilk or all of the different types of infant formula, meaning that nutrients such as calcium from breastmilk and infant formula may be misrepresented. Given the high percentage of young children receiving breastmilk and infant formula, there is an urgent need to develop dietary assessment tools that incorporate breastmilk and a range of infant formula nutrient analyses. Lastly, the young age of this cohort limits the availability of appropriate NRVs for macronutrients and micronutrients (Table 4).

The mothers of these children generally had a high socio-economic status, high levels of education, and were of Caucasian ethnicity, which limits the generalizability of the findings. Participants were from high-income families, a factor known to be predictive of healthy eating patterns [33]. Additionally, mothers who agree to participate in research are often better informed about the importance of infant nutrition than the general population [24], which may explain why the children in this study largely met dietary guidelines and nutrient reference value recommendations.

## 5. Conclusions

This study provides, for the first time, a snapshot of the dietary intake of a cohort of young children aged one year in Western Australia. Results are presented based on breastfeeding status. The cohort met NRV cut-offs for most micronutrients; however, they consumed inadequate iodine (breastfed group) and exceeded sodium recommendations. The quality of the diet based on the food group intake did not meet recommendations, with the children over-consuming fruit and discretionary foods while under-consuming vegetables and cereals and grains foods. These findings highlight areas for potential improvement in the dietary intake of one-year-old children. Specific focus should be on reducing the intake of discretionary foods and providing children with opportunities to consume more vegetable and cereal-based foods. Reducing fruit intake may allow for consumption of foods from other food groups. Addressing these imbalances could help guide future dietary recommendations and interventions to support healthier eating habits and better nutritional outcomes for young children. 

## Figures and Tables

**Table 1:** Characteristics of ORIGINS mothers and their children.

	*n*	
**Child characteristics**		
Age (months): mean ± SD	779	12.3 ± 0.5
Plurality: *n* (%)	779	
Singleton		769 (98.7%)
Twins		10 (1.3%)
Sex: *n* (%)		
Male	779	400 (51.4%)
Female		379 (48.6%)
Gestational age (weeks): mean ± SD	779	39.3 ± 1.1
Birth weight (g): mean ± SD	733	3432.6 ± 465.2
Birth length (cm): mean ± SD	733	50.6 ± 2.4
Current weight (kg): mean ± SD	771	10.0 ± 1.4
Current height (cm): mean ± SD	757	75.0 ± 4.5
Attendance at Child Care: *n* (%)	779	
Yes		265 (34.0%)
No		514 (66.0%)
**Maternal characteristics**		
Age at recruitment (years) ^a^: mean ± SD	774	32.1 ± 4.3
Parity ^a^: *n* (%)	700	
0		423 (60.4%)
1		198 (28.3%)
2		63 (9.0%)
3+		16 (2.3%)
Ethnic origin ^b^: *n* (%)	745	
Caucasian		625 (83.9%)
Aboriginal not Torres Strait Islander		2 (0.3%)
African		2 (0.3%)
Asian		59 (7.9%)
Polynesian		2 (0.3%)
Other		55 (7.4%)
Born in Australia ^b^: *n* (%)	683	
Yes		388 (56.8%)
No		295 (43.2%)
Pre-pregnancy BMI (kg/m^2^) ^a^: median (IQR)	760	24.6 (21.9, 28.7)
Level of education ^c^: *n* (%)	683	
High school or lower		144 (21.1%)
Trade/Apprenticeship/Diploma		142 (20.8%)
University Degree		397 (58.1%)
Employment status (1 year) ^a^: *n* (%)	773	
Parent/home duties/unemployed		273 (35.3%)
Employed full-time (paid or unpaid)		103 (13.3%)
Employed part-time (paid or unpaid)		310 (40.1%)
Student		15 (1.9%)
Other		72 (9.3%)
**Family characteristics**		
Household income (1 year) ^a^: *n* (%)	721	
Up to AUD 50,000 per year		54 (7.5%)
AUD 50,001 to AUD 75,000 a year		62 (8.6%)
AUD 75,001 to AUD 100,000 a year		139 (19.3%)
AUD 100,001 to AUD 150,000 a year		264 (36.6%)
More than AUD 150,000 a year		202 (28.0%)
Household composition—number of adults ^a^: *n* (%)	771	
1		23 (3.0%)
2		676 (87.7%)
3+		72 (9.3%)
Household composition—number of children ^a^: *n* (%)	770	
1		453 (58.8%)
2		222 (28.8%)
3+		95 (12.3%)

Data are presented as observed counts and percentages, mean ± standard deviation (SD), or median (interquartile range). ^a^ Characteristics are by pregnancy, as these may change with subsequent pregnancies. ^b^ Characteristics are by mother. ^c^ Level of education did not change with subsequent pregnancies for any of the participants.

**Table 2:** Summary of breastfeeding, infant formula feeding, and cow’s milk intake of ORIGINS children at one year.

	*n*	
**Breastfeeding intake**		
Ever breastfed: *n* (%)	779	
Yes		731 (93.8%)
No		48 (6.2%)
Exclusively breastfed (to 6 months of age) ^a^: *n* (%)	779	
Yes		170 (21.8%)
No		609 (78.2%)
Breastfeeding at one year: *n* (%)	779	
Yes		323 (41.5%)
No		456 (58.5%)
Duration of breastfeeding (months) ^b^: mean ± SD	408	5.2 ± 3.5
Frequency of breastfeeding (feeds per day at one year): median (IQR)	221	3 (2, 4)
**Formula feeding intake**		
Ever fed infant formula milk: *n* (%)	779	
Yes		618 (79.3%)
No		161 (20.7%)
Age formula milk first introduced (months): median (IQR)	609	0.5 (0, 4)
Exclusive formula feeding (to 6 months of age) ^c^: *n* (%)	779	
Yes		48 (6.2%)
No		731 (93.8%)
Formula feeding at one year: *n* (%)	779	
Yes		452 (58.0%)
No		327 (42.0%)
Frequency of formula feeding (feeds per day at one year): median (IQR)	449	3 (2, 4)
**Cow’s milk intake**		
Ever fed cow’s milk (for a feed): *n* (%)	779	
Yes		338 (43.4%)
No		441 (56.6%)
Frequency of cow’s milk (feeds per day at one year): median (IQR)	338	1 (1, 2)

Data are presented as observed counts and percentages, mean ± standard deviation (SD), or median (interquartile range). ^a^ This means all feeds are breastmilk, i.e., no infant formula or cow’s milk. ^b^ This is the duration of breastfeeding for those who did breastfeed and are not still breastfeeding at one year (i.e., does not include those who never breastfed or those who are still breastfeeding). ^c^ This means all feeds are infant formula, i.e., no breastmilk or cow’s milk.

**Table 3:** Summary of ORIGINS children’s introduction to solid foods.

	*n*	
Solid foods introduced by one year: *n* (%)	729	
Yes		727 (99.7%)
No		2 (0.3%)
Age of introduction to solids: *n* (%)	729	
<6 months		79 (10.8%)
≥6 months		650 (89.2%)
Child eats all food you eat: *n* (%)	738	
Yes		662 (89.7%)
No		42 (5.7%)
Sometimes		34 (4.6%)
Child is a fussy eater: *n* (%)	738	
Yes		83 (11.2%)
No		578 (78.3%)
Sometimes		77 (10.4%)
Child has been exposed to the following foods: *n* (%)	743	
Eggs (and egg products)		733 (98.7%)
Cow’s milk (and milk products)		727 (97.8%)
Wheat (and wheat products)		739 (99.5%)
Fish		703 (94.6%)
Peanut		682 (91.8%)
Cashew nut		383 (51.6%)
Presence of food allergy: *n* (%)	743	
Yes		64 (8.6%)
No		679 (91.4%)

Data are presented as observed counts and percentages.

**Table 4:** Daily energy and macronutrient intake of the ORIGINS children at one year, stratified by breastfeeding status.

Nutrient	Breastfed *n* = 239			Non-Breastfed *n* = 299					
	Daily Intake	NRV ^a^ Compliance *n* (%)		Daily Intake	NRV ^a^ Compliance *n* (%)		NRV ^a^		
		<EAR ^b^	>AI ^c^		<EAR ^b^	>AI ^c^	EER ^d^	EAR ^b^	AI ^c^
Energy—Including fibre (kJ)									
All participants	3286.0 ± 807.0	-	-	3612.1 ± 748.0	-	-	-	-	-
Males	3456.7 ± 826.6	-	-	3654.7 ± 787.3	-	-	3500	-	-
Females	3122.4 ± 755.4	-	-	3552.8 ± 688.1	-	-	3200	-	-
Protein (g)	39.4 ± 11.5	1 (0.4%)	-	41.7 ± 10.9	0 (0.0%)		-	12	-
Fat (g)									
Total ^e^	23.5 ± 8.0		61 (25.5%)	28.7 ± 7.2		131 (43.8%)	-	-	30
Saturated	9.8 ± 4.2			11.7 ± 4.3			-	-	-
Polyunsaturated	3.2 ± 1.0			3.0 ± 1.1			-	-	-
Monounsaturated	7.9 ± 2.7			8.3 ± 2.5			-	-	-
Carbohydrate (g)									
Total ^e^	97.2 ± 26.3		126 (52.7%)	103.9 ± 23.7		195 (65.2%)	-	-	95
Sugar	44.4 ± 15.2			47.6 ± 14.7			-	-	-
Starch	50.5 ± 18.5			47.4 ± 15.5			-	-	-
Fibre ^f^	12.9 ± 4.0	-	92 (38.5%)	11.3 ± 3.4		73 (24.4%)	-	-	14

Data are presented as observed counts and percentages or mean ± standard deviation (SD). ^a^ NRV, Nutrient Reference Values, adapted from Nutrient Reference Values for Australian and New Zealand Including Recommended Dietary Intakes (https://www.nhmrc.gov.au/about-us/publications/nutrient-reference-values-australia-and-new-zealand-including-recommended-dietary-intakes, accessed on 2 July 2024). Copyright 2016 by the Commonwealth of Australia. ^b^ EAR, Estimated Average Requirement for children aged 1–3 years. ^c^ AI, Adequate Intake. ^d^ EER, Estimated Energy Requirements for children 12 months of age. ^e^ The AI is for infants aged 7–12 months. ^f^ The AI is for children aged 1–3 years.

**Table 5:** Daily micronutrient intake of the ORIGINS children at one year, stratified by breastfeeding status.

Nutrient	Breastfed *n* = 239				Non-Breastfed *n* = 299						
	Daily Intake	NRV ^a^ Compliance *n* (%)			Daily Intake	NRV ^a^ Compliance *n* (%)			NRV ^a^		
		<EAR ^b^	>AI ^c^	>UL ^d^		<EAR ^b^	>AI ^c^	>UL ^d^	EAR ^b^	AI ^c^	UL ^d^
Vitamin C (mg)	75 (55, 102)	3 (1.3%)	-	-	86 (64, 108)	2 (0.7%)	-	-	25	-	-
Folate (µg) ^e^	157 (126, 194)	48 (20.1%)	-	1 (0.4%)	175 (142, 209)	30 (10.0%)	-	1 (0.3%)	120	-	300
Retinol Equivalents (µg)	357 (287, 457)	13 (5.4%)	-	16 (6.7%)	421 (346, 501)	4 (1.3%)	-	23 (7.7%)	210	-	600
Magnesium (mg) ^f^	169 (143, 193)	0 (0.0%)	-	NA	175 (139, 198)	0 (0.0%)	-	NA	65	-	65
Phosphorus (mg)	625 (480, 769)	19 (7.9%)	-	0 (0.0%)	672 (550, 840)	8 (2.7%)	-	0 (0.0%)	380	-	3000
Calcium (mg)	392 (257, 525)	101 (42.3%)	-	0 (0.0%)	559 (439, 677)	33 (11.0%)	-	0 (0.0%)	360	-	2500
Iron (mg)	5 (4, 6)	42 (17.6%)	-	0 (0.0%)	7 (6, 8)	9 (3.0%)	-	0 (0.0%)	4	-	20
Zinc (mg)	5 (4, 6)	6 (2.5%)	-	14 (5.9%)	6 (5, 7)	1 (0.3%)	-	51 (17.1%)	2.5	-	7
Sodium (mg) ^g^	678 (500, 847)	-	212 (88.7%)	21 (8.8%)	685 (557, 833)	-	275 (92.0%)	21 (7.0%)	-	200–400	1000
Iodine (mg)	41 (31, 64)	180 (75.3%)	-	0 (0.0%)	66 (52, 86)	146 (48.8%)	-	3 (1.0%)	65	-	200
Potassium (mg)	1540 (1234, 1785)	-	36 (15.1%)	-	1684 (1359, 1950)	-	62 (20.7%)	-	-	2000	-

Data are presented as observed counts and percentages or median (interquartile range). ^a^ NRV, Nutrient Reference Values, adapted from Nutrient Reference Values for Australia and New Zealand Including Recommended Dietary Intakes (https://www.nhmrc.gov.au/about-us/publications/nutrient-reference-values-australia-and-new-zealand-including-recommended-dietary-intakes, accessed on 2 July 2024). Copyright 2016 by the Commonwealth of Australia. ^b^ EAR, Estimated Average Requirement for children aged 1–3 years. ^c^ AI, Adequate Intake for children aged 1–3 years. ^d^ UL, upper limit of intake for children aged 1–3 years. ^e^ The UL for folate is for intake from fortified foods and supplements as folic acid. ^f^ The UL listed for magnesium refers to supplements. ^g^ The AI for sodium is assumed to be 400 mg in determining the compliance proportions. If 200 mg was used, then compliance (>AI) would be *n* = 237 (99.2%) for breastfed children and *n* = 299 (99.7%) for those non-breastfed.

**Table 6:** Daily intake and servings of the five food groups and discretionary foods consumed by the ORIGINS children at one year, stratified by breastfeeding status.

Food Group	Breastfed *n* = 323		Non-Breastfed *n* = 456		Recommendations	
	Daily Intake (g)	Serves/Day ^a^	Daily Intake (g)	Serves/Day ^a^	AGHE Serving Size	Recommended Serves/Day
Fruit	223 (156, 304)	1.5 (1.1, 2.1)	199 (133, 289)	1.3 (0.9, 2.0)	150 g	0.5
Vegetables	95 (71, 134)	1.3 (0.9, 1.8)	96 (67, 127)	1.3 (0.9, 1.7)	75 g	2–3
Cereals and grains foods, mostly wholegrain	113 (77, 145)	1.8 (1.3, 2.6)	107 (77, 139)	1.8 (1.3, 2.4)	40 g bread	4
Dairy foods, i.e., milk, yoghurt, cheese, and/or alternatives (mostly reduced fat)	125 (52, 295)	0.9 (0.4, 1.6)	431 (304, 569)	1.9 (1.5, 2.6)	200 g yoghurt	1–1.5
Lean meat and poultry, fish, eggs, nuts, and seeds	89 (59, 114)	1.2 (0.8, 1.6)	88 (62, 121)	1.2 (0.8, 1.7)	80 g lean poultry	1
Discretionary foods	22 (9, 42)		26 (12, 47)		418 kJ	0

Data are presented as median (interquartile range). ^a^ Serves per day estimated using the Australian Guide to Healthy Eating (National Health and Medical Research Council & Department of Health and Aging. (2013). Australian Dietary Guidelines; National Health and Medical Research Council: Canberra, Australia, accessed on 19 July 2024).

## Data Availability

The data presented in this study are available on request from the corresponding author due to privacy reasons.

## Supplementary Materials

The following supporting information can be downloaded at: https://www.mdpi.com/article/10.3390/nu17091566/s1. Figure S1: Flow chart showing inclusion.
