# Supplementary figures and images for: ORIGINS: Nutritional Profile of Children Aged One Year in a Longitudinal Birth Cohort
Source: Nutrients. 2025 May 1;17(9):1566. doi: 10.3390/nu17091566 (PMC12073640; doi:10.3390/nu17091566)

## Supplementary Figure S1

Supplementary Figure S1. Flow chart showing inclusion.

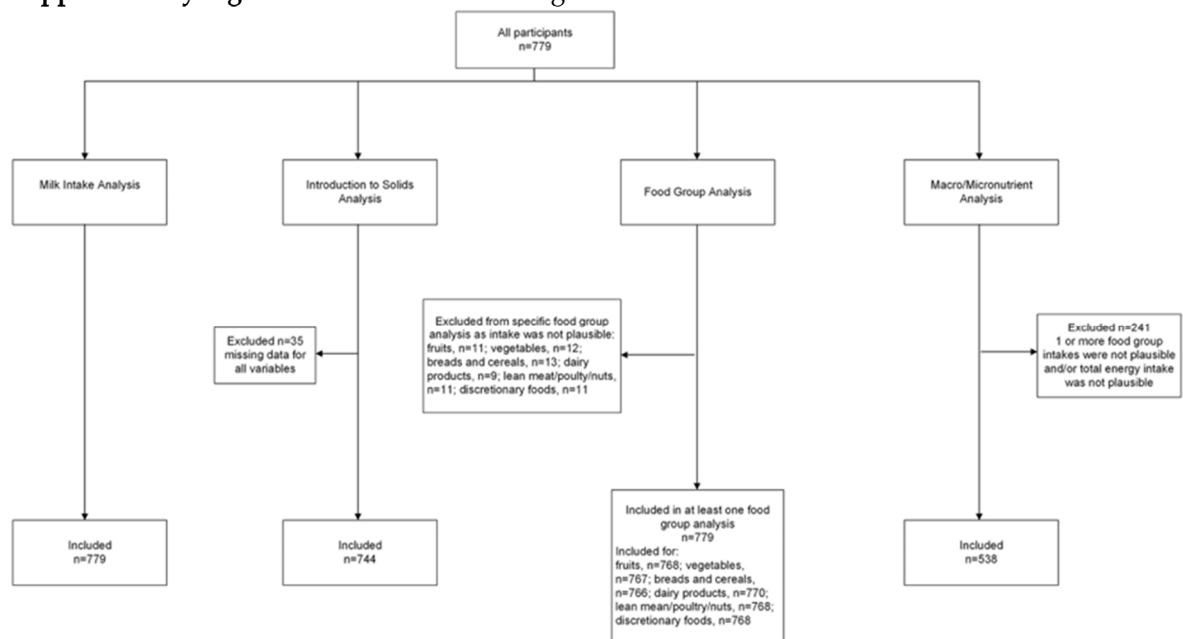

Supplement: Supplementary file 1 [file nutrients-17-01566-s001.zip › nutrients-3576827-supplementary.pdf]
